# Machine Learning Approach on Predictive Model Establishment for In-Hospital Mortality in Acute Myocardial Infarction Patients Post-Percutaneous Coronary Intervention: Solutions for Databases With Dimensionality Reduction and Class Imbalance

**DOI:** 10.31083/RCM39271

**Published:** 2025-09-25

**Authors:** Wenqiang Li, Peng Lei, Rongyan Dong, Shilin He, Zheng Zhang, Bing Han

**Affiliations:** ^1^The First School of Clinical Medical, Lanzhou University, 730000 Lanzhou, Gansu, China; ^2^Department of Cardiology, Qinghai Provincial People's Hospital, 810007 Xining, Qinghai, China; ^3^Department of Cardiology, The First Hospital of Lanzhou University, 730013 Lanzhou, Gansu, China; ^4^Department of Cardiology, Tianshui Chinese Medicine Hospital, 741000 Tianshui, Gansu, China

**Keywords:** in-hospital mortality, acute myocardial infarction, machine learning, prediction model, data imbalance, dimensionality reduction

## Abstract

**Background:**

Acute myocardial infarction (AMI) remains a leading cause of mortality and disability globally. Although percutaneous coronary intervention (PCI) has significantly reduced in-hospital mortality (IHM), the resultant class imbalance complicates accurate risk prediction. While machine learning (ML) demonstrates potential in predicting IHM, there is a lack of models that provide both high accuracy and personalized risk assessment.

**Methods:**

This retrospective study was conducted at the First Hospital of Lanzhou University from January 1, 2019, to December 31, 2020. We employed three data processing methods: synthetic minority over-sampling technique (SMOTE), Boruta, and grid search cross-validation (GSCV). Subsequently, six ML algorithms were implemented. Model performance was evaluated using accuracy, sensitivity, precision, F1-score, area under the receiver operating characteristic curve (AUROC), and area under the precision-recall curve (AUPRC).

**Results:**

The study cohort consisted of 1693 patients diagnosed with AMI, of whom 34 (2.0%) experienced IHM following PCI. After employing SMOTE to balance the dataset, 32 independent risk factors were identified using the Boruta feature selection method. Among the evaluated ML models, ensemble algorithms demonstrated superior performance. For instance, the Light Gradient-Boosting Machine (LightGBM) framework achieved a predictive accuracy with an AUROC of 0.93 (95% confidence interval (CI): 0.82–1.00) and an AUPRC of 0.62 (95% CI: 0.17–0.96). Additional performance metrics included an accuracy of 0.988, a precision of 0.625, a sensitivity of 0.625, a specificity of 0.994, and an F1-score of 0.625.

**Conclusion:**

Utilizing SMOTE for class balancing, Boruta for feature selection, GSCV for optimal hyperparameter tuning, and LightGBM for model development achieved strong predictive performance for IHM following AMI. These findings underscore the significance of robust processing and careful algorithm selection.

## 1. Introduction

Acute myocardial infarction (AMI) continues to be a leading cause of mortality and disability globally, particularly in middle- and high-income countries [1]. The in-hospital mortality (IHM) rate for AMI is influenced by various factors, including the quality of healthcare, patient comorbidities, and the timeliness of treatment [2]. Recent advances in emergency care, the establishment of chest pain centers, and the widespread adoption of percutaneous coronary intervention (PCI) have collectively contributed to a reduction in IHM rates.

To support clinical decision-making, several risk stratification tools—such as the Thrombosis In Myocardial Infarction (TIMI), Global Registry of Acute Coronary Events (GRACE), and Primary Angioplasty in Myocardial Infarction (PAMI) scores—are commonly employed to classify patients by risk level and guide treatment strategies [3, 4, 5, 6]. However, most of these systems were developed over a decade ago and are implicitly based on an ‘average patient’, which limits their ability to provide individualized probability estimates. Models based on machine learning (ML) have demonstrated significantly superior performance compared to traditional statistical methods [7, 8, 9, 10]. Aziz *et al*. [11] utilized ML to predict both short- and long-term mortality in patients with ST-Elevation Myocardial Infarction (STEMI) and compared its efficacy with traditional risk scores. In a multi-ethnic cohort, ML models outperformed the TIMI score in risk classification. Shakhgeldyan *et al*. [12] developed IHM prediction models using multivariate logistic regression (MLR), random forest (RF), and stochastic gradient boosting (SGB), all of which surpassed the GRACE score in predictive accuracy. Although prior studies have shown that ML models outperform traditional risk scores [13, 14], they still lack the capability to provide individualized predictions for specific patients. D’Ascenzo *et al*. [15] created the PRAISE score to estimate one-year mortality in patients with Acute Coronary Syndrome (ACS), which accurately predicts the risk of all-cause mortality, myocardial infarction (MI), and major bleeding (MB). While this score provides both event probabilities and risk stratification, it does not quantify the contribution of patient-specific factors to these outcomes.

The objective of this study was to develop predictive models for IHM in patients with AMI following PCI, thereby enabling personalized risk assessment and quantifying the impact of individual variables. The key contributions of this research include: (1) demonstrating the influence of data processing strategies and ML algorithms on model performance; (2) emphasizing the importance of robust data processing and algorithm selection; (3) illustrating that in highly imbalanced datasets, the area under the precision-recall curve (AUPRC) offers greater discriminative power than the area under the receiver operating characteristic curve (AUROC); and (4) developing a high-performing model enhanced by SHapley Additive exPlanation (SHAP) analysis to visualize feature contributions, thereby improving interpretability and supporting individualized clinical decision-making.

## 2. Materials and Methods

### 2.1 Study Population

**Fig. 1.**
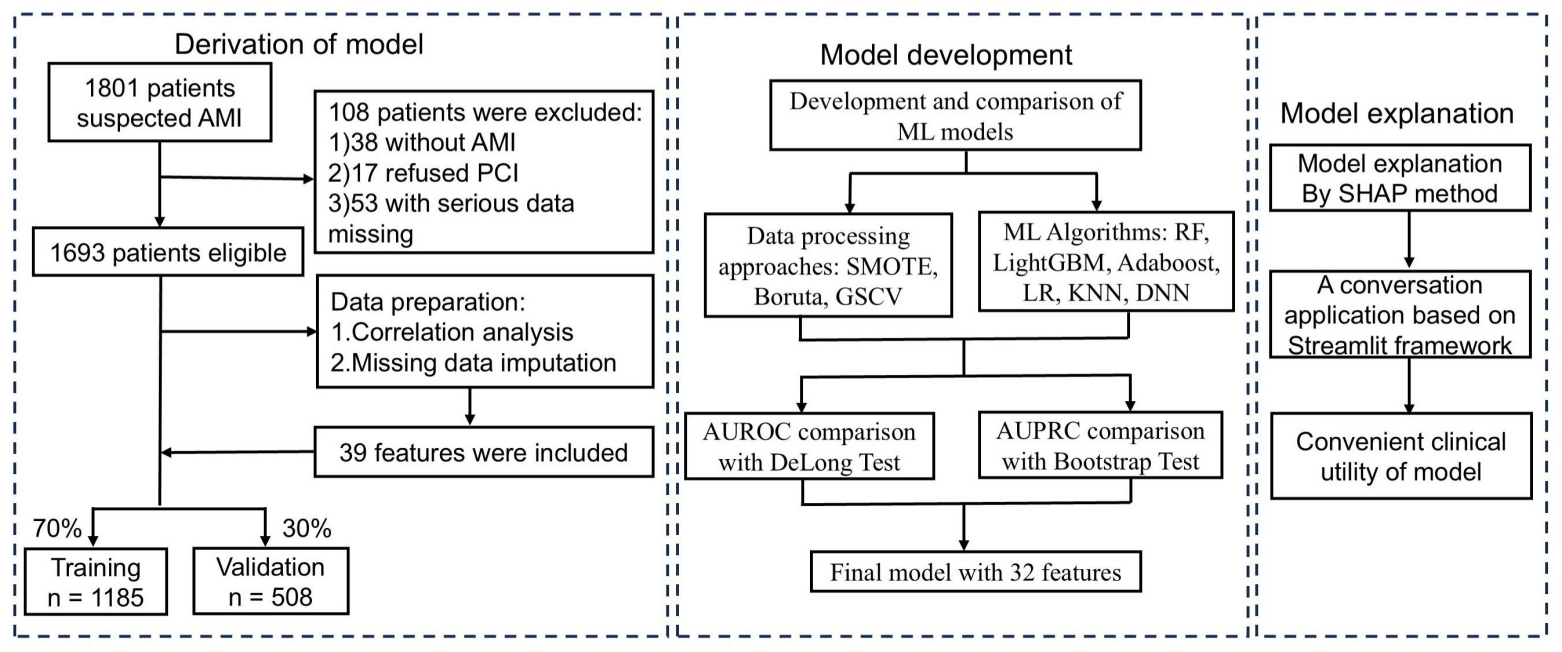
**Flowchart of AMI patient inclusion and exclusion, and the ML model development process**. AMI, acute myocardial infarction; PCI, percutaneous coronary intervention; ML, machine learning; SMOTE, synthetic minority over-sampling technique; GSCV, grid search with cross-validation; SHAP, shapley additive explanation; AUROC, area under the receiver operating characteristic curve; AUPRC, area under the precision-recall curve; RF, Random Forest; LightGBM, Light Gradient Boosting Machine; AdaBoost, Adaptive Boosting; LR, Logistic Regression; KNN, K-Nearest, Neighbors; DNN, Deep Neural Networks.

This retrospective study was conducted at the First Hospital of Lanzhou University in Gansu Province, spanning from January 1, 2019, to December 31, 2020. The study included all consecutive hospitalized patients diagnosed with AMI who received PCI. The diagnosis of AMI was established in accordance with the European Society of Cardiology Guidelines [2]. The inclusion criteria were as follows: (1) a confirmed diagnosis of AMI, (2) age ≥18 years, (3) patients who underwent PCI, and (4) availability of complete clinical information. The inclusion and exclusion processes are illustrated in Fig. 1. Initially, 1801 patients were identified; however, 53 were excluded due to significant missing data, 38 due to misdiagnoses, and 17 because they did not undergo PCI. Ultimately, 1693 patients were included for further analysis.

### 2.2 Data Collection

Clinical and laboratory data for patients with AMI were extracted from electronic medical records, with a particular focus on IHM. The relevant laboratory tests and clinical data are summarized in Table 1. The results of the initial laboratory test conducted upon hospital admission prior to PCI were recorded, along with the corresponding blood sample data. To manage missing data, two approaches were employed: features with more than 20% missing data were excluded, while for those with less than 20% missing data, the IterativeImputer method was utilized to minimize bias. Based on parameters from established risk scores (e.g., GRACE, TIMI) and the latest guideline-based insights into AMI pathophysiology, supplemented by input from clinical experts, we identified 39 potential prognostic variables for AMI outcomes [1, 3, 5]. These variables, encompassing demographic information, cardiovascular history, and laboratory indicators, were incorporated into the analysis. Specifically, the variables included gender, age, total ischemia time (TIT), type 2 diabetes mellitus (T2DM), hypertension (HTN), hyperlipidemia (HLD), peripheral artery disease (PAD), smoking history (SH), coronary artery disease (CAD), bleeding history (HB), body mass index (BMI), systolic blood pressure (SBP), diastolic blood pressure (DBP), heart rate (HR), creatinine (CREA), uric acid (UA), random blood glucose (RBG), low-density lipoprotein cholesterol (LDL-C), estimated glomerular filtration rate (eGFR), hematocrit (HCT), neutrophil count (NEUT), lymphocyte count (LYMPH), neutrophil-to-lymphocyte ratio (NLR), hemoglobin (HGB), platelet count (PLT), C-reactive protein (CRP), myoglobin (MYO), creatine kinase–myocardial band (CK-MB), troponin I (TNI), N-terminal pro–B-type natriuretic peptide (NT-proBNP), hemoglobin A1c (HbA1c), left atrial diameter (LAD), left ventricular ejection fraction (LVEF), left ventricular end-diastolic volume (LVEDV), left ventricular end-systolic volume (LVESV), MB, ventricular fibrillation (VF), atrial fibrillation (AF), and cardiogenic shock (CS).

**Table 1.** **Comparison of demographic and clinical characteristics**.

Variables		Survived (n = 1659)	Deceased (n = 34)	Training (n = 1185)	Testing (n = 508)
	Age (y)	60.85 ± 11.06	65.47 ± 13.29*	60.88 ± 11.19	66.1 ± 10.98
	Gender (%)	1405 (84.69)	27 (79.41)	1005 (84.811)	427 (84.06)
	TIT (h)	40.84 ± 89.22	44.24 ± 67.12	38.69 ± 69.22	46.08 ± 122.88
Medical history					
	T2DM (%)	311 (18.75)	14 (41.18)**	222 (18.73)	103 (20.28)
	HTN (%)	765 (46.11)	17 (50)	531 (44.81)	251 (49.41)
	HLD (%)	402 (24.23)	2 (5.88)*	283 (23.88)	121 (23.82)
	PAD (%)	73 (4.4)	2 (5.88)	52 (4.39)	23 (4.53)
	SH (%)	840 (50.63)	12 (35.29)	595 (50.21)	257 (50.59)
	CAD (%)	154 (9.28)	4 (11.76)	112 (9.45)	46 (9.06)
	BH (%)	33 (1.99)	0 (0)	21 (1.77)	12 (2.36)
	BMI (Kg/m^2^)	23.89 ± 3.39	23.4 ± 3.45	23.81 ± 3.42	24.04 ± 3.31
Baseline vital signs					
	SBP (mmHg)	116.99 ± 25.15	93.59 ± 27.85***	115.83 ± 25.69	118.13 ± 24.69
	DBP (mmHg)	74.6 ± 15.63	62.21 ± 18.36***	74 (62, 83)	76 (66, 83)
	HR (beats/min)	80 (69, 90)	89.5 (78.75, 100.75)**	81.24 ± 17.9	81.01 ± 17.71
Baseline laboratory values					
	CREA (umol/L)	72 (63, 84)	114 (88, 143)***	81.26 ± 61.26	80.73 ± 54.64
	UA (umol/L)	354.24 ± 102.31	448.68 ± 122.18***	356.6 ± 103.19	355.07 ± 104.52
	RBG (mmol/L)	6.78 (5.56, 9.08)	12.08 (7.04, 17.37)***	8.26 ± 4.4	8.3 ± 4.82
	LDLC (mmol/L)	2.94 ± 0.89	2.85 ± 0.92	2.91 ± 0.9	2.98 ± 0.87
	eGFR (mL/min/1.73 m^2^)	90.76 (71, 112)	52.9 (40.67, 67.98)***	91.76 ± 33.1	92.37 ± 32.76
	CRP (mg/L)	9.85 (3.31, 28.1)	53.85 (12.7, 92.8)***	27.85 ± 45.31	25.04 ± 43.43
	HCT (%)	44.58 ± 6.02	43.52 ± 6.61	44.46 ± 5.9	44.79 ± 6.33
	NEUT (10^9^/L)	8.17 ± 5.6	10.51 ± 3.79*	8.17 ± 5.6	8.33 ± 5.54
	LYMPH (10^9^/L)	1.57 ± 1.4	1.6 ± 1.14	1.56 ± 1.39	1.59 ± 1.43
	NLR	7.21 ± 7.41	9.97 ± 10.4*	7.3 ± 7.73	7.18 ± 6.91
	HGB (g/L)	151.13 ± 19.57	146.38 ± 21.78	150.85 ± 19.48	151.47 ± 19.95
	PLT (10^9^/L)	190.38 ± 66.26	182.56 ± 65.77	185 (150, 222)	184 (145, 229)
	MYO (ng/mL)	403.76 ± 331.51	598.83 ± 339.33**	412.11 ± 334.88	397.33 ± 327.61
	CKMB (ng/mL)	137.38 ± 160.58	162.98 ± 177.22	139.53 ± 162.29	134.1 ± 157.74
	TNI (ng/mL)	2 (0.39, 7.85)	7.45 (1.18, 15.75)***	6.16 ± 8.32	6.11 ± 8.31
	NT-proBNP (pg/mL)	560 (207, 1715)	3820 (1305, 7338)***	1910.57 ± 3954.32	2154.48 ± 4703.93
	HbA1c (%)	5.8 (5.4, 6.4)	6.42 (5.53, 9.41)***	6.31 ± 1.6	6.33 ± 1.57
Echocardiographic findings					
	LAD (cm)	3.28 ± 0.4	3.32 ± 0.59	3.29 ± 0.39	3.27 ± 0.43
	LVEF (%)	53 (48, 57)	44 (35.75, 48.26)***	51.75 ± 7.49	51.84 ± 7.52
	LVEDV (mL)	128.6 ± 34.58	129.91 ± 35.01	128.12 ± 34.87	129.81 ± 33.88
	LVESV (mL)	62.44 ± 24.30	76.19 ± 25.77**	62.42 ± 24.63	63.39 ± 23.86
In-hospital complications					
	MB (%)	14 (0.84)	4 (11.76)***	12 (1.01)	6 (1.18)
	VF (%)	32 (1.93)	11 (32.35)***	30 (2.53)	13 (2.56)
	AF (%)	25 (3.94)	7 (18.42)**	49 (4.14)	16 (3.15)
	CS (%)	59 (3.56)	6 (17.65)***	35 (2.95)	22 (4.33)
	Deceased (%)	0 (0)	34 (100)	24 (2.03)	10 (1.97)

Compared to Survived, * *p* < 0.05; ** *p* < 0.01; *** *p* < 0.001. Continuous values are presented as median ± standard deviation, while categorical values are expressed as number (percentage). The eGFR was calculated using the first available serum creatinine measurement obtained within the first 24 hours following admission. TIT, total ischemia time; T2DM, Type 2 Diabetes Mellitus; HTN, hypertension; HLD, hyperlipidemia; PAD, peripheral artery disease; SH, smoking history; CAD, coronary artery disease; HB, bleeding history; BMI, body mass index; SBP, systolic blood pressure; DBP, diastolic blood pressure; HR, heart rate; CREA, creatinine; UA, uric acid; RBG, random blood glucose; LDL-C, low-density lipoprotein cholesterol; eGFR, estimated glomerular filtration rate; HCT, hematocrit; NEUT, neutrophil; LYMPH, lymphocyte; NLR, neutrophil lymphocyte ratio; HGB, hemoglobin; PLT, platelet count; CRP, serum C-reactive protein; MYO, myoglobin; CK-MB, creatine kinase-myocardial band; TNI, troponin I; NT-proBNP, n-terminal pro-B-type natriuretic peptide; HbA1c, hemoglobin A1c; LAD, left atrial diameter; LVEF, left ventricular ejection fraction; LVEDV, left ventricular end-diastolic volume; LVESV, left ventricular end-systolic volume; MB, major bleeding; VF, ventricular fibrillation; AF, atrial fibrillation; CS, cardiogenic shock.

In this study, CAD was defined as the presence of ≥50% stenosis, prior revascularization, or a history of MI. MB was characterized by a hemoglobin drop of ≥3 g/dL or the transfusion of ≥2 units of blood. CS was diagnosed based on a SBP of less than 90 mmHg persisting for over 30 minutes, the necessity for vasopressors or mechanical support, evidence of hypoperfusion, or classification as Killip class IV. HB was defined as any clinically significant bleeding occurring within the previous 12 months.

### 2.3 Model Development and Comparison

Patients were randomly divided into training and testing cohorts in a 70:30 ratio. Three data processing techniques were employed: Synthetic Minority Over-sampling Technique (SMOTE) to address class imbalance [16, 17, 18], Boruta for feature selection [19], and Grid Search with Cross-Validation (GSCV) for hyperparameter tuning [20]. Six ML algorithms were evaluated: Random Forest (RF), Light Gradient Boosting Machine (LightGBM), Adaptive Boosting (AdaBoost), Logistic Regression (LR), K-Nearest Neighbors (KNN), and Deep Neural Networks (DNN).

Model development adhered to a structured framework: (1) identifying optimal processing methods, (2) selecting the best-performing algorithm, and (3) constructing the final predictive model by integrating the chosen strategies (Fig. 1). Model performance was primarily assessed using the AUROC and AUPRC [21], while secondary metrics such as accuracy, precision, sensitivity, specificity, and F1-score supported model selection. To enhance interpretability, SHAP values were employed to assess feature importance and quantify each variable’s contribution to the model’s predictions.

### 2.4 Statistical Analysis

Baseline characteristics between the survival and mortality groups were compared. For normally distributed data, the Independent Samples *t*-test was employed, with results expressed as the mean ± standard deviation. For non-normally distributed data, the Mann–Whitney U test was utilized, and results were presented as the median ± interquartile range. DeLong’s test was conducted to compare AUROC differences between models, while the Bootstrap test assessed differences in AUPRC. Categorical variables were analyzed using the Chi-Squared test. All statistical analyses were performed using R 4.3.1 software (R Foundation for Statistical Computing, Vienna, Austria), whereas ML algorithms, including BorutaShap, SHAP, AUROC, and AUPRC, were implemented using Python 3.11.9 software (Python Software Foundation, Wilmington, DE, USA). A two-tailed *p*-value of <0.05 was deemed statistically significant.

## 3. Results

### 3.1 Population Characteristics

This prospective study included 1693 patients in the derivation cohort for the development of the IHM prediction model. Among these patients, 34 (2.0%) died in-hospital following PCI. A detailed comparison of demographic and clinical variables among the training, testing, survived, and deceased cohorts is provided in Table 1.

### 3.2 Model Selection and Performance Comparison

**Fig. 2.**
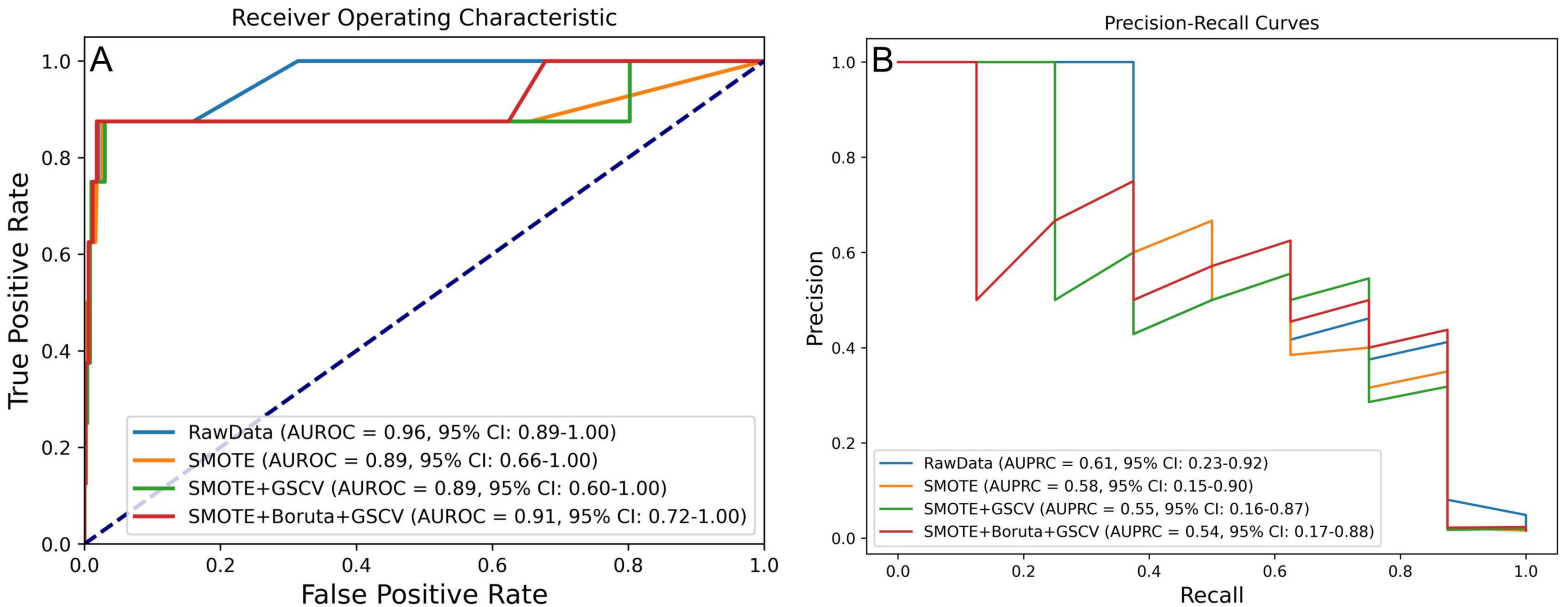
**Model performance under four processing strategies for predicting IHM in AMI patients post-PCI**. (A) AUROC and (B) AUPRC values across different processing levels. IHM, in-hospital mortality.

The first phase centered on optimizing data processing while consistently employing the RF algorithm. This process sequentially applied the SMOTE for class balancing, GSCV for hyperparameter tuning, and the Boruta algorithm for feature selection, with the aim of identifying the most effective processing strategy. Model performance was assessed using the AUROC and the AUPRC, as illustrated in Fig. 2A,B.

DeLong and bootstrap tests were employed to compare the AUROC and the AUPRC across various processing strategies (**Supplementary Fig. 1**). The results indicated no statistically significant differences (*p* > 0.05), suggesting that the processing method alone did not substantially impact model performance. Consequently, model selection was guided by secondary metrics and a preference for simpler models. For the model trained using SMOTE, Boruta, and GSCV, the validation set yielded an AUROC of 0.91 (95% CI: 0.72–1.00) and an AUPRC of 0.54 (95% CI: 0.17–0.88).

Additional metrics, including accuracy, precision, sensitivity, specificity, and F1 score, were utilized for model evaluation. As summarized in Table 2, the model trained with SMOTE and GSCV achieved the best overall performance, with an AUROC of 0.892, accuracy of 0.986, precision of 0.556, sensitivity of 0.625, specificity of 0.992, and an F1 score of 0.588.

**Table 2.** **Data processing effects on IHM prediction performance in AMI patients post-PCI**.

Data processing approaches	Accuracy	Precision	Sensitivity	Specificity	F1-score	AUROC	AUPRC
RawData	0.986	1.000	0.125	1.000	0.222	0.964	0.611
SMOTE	0.984	0.500	0.500	0.992	0.500	0.889	0.577
SMOTE + GSCV	0.986	0.556	0.625	0.992	0.588	0.892	0.548
SMOTE + Boruta + GSCV	0.986	0.571	0.500	0.994	0.533	0.913	0.543

**Fig. 3.**
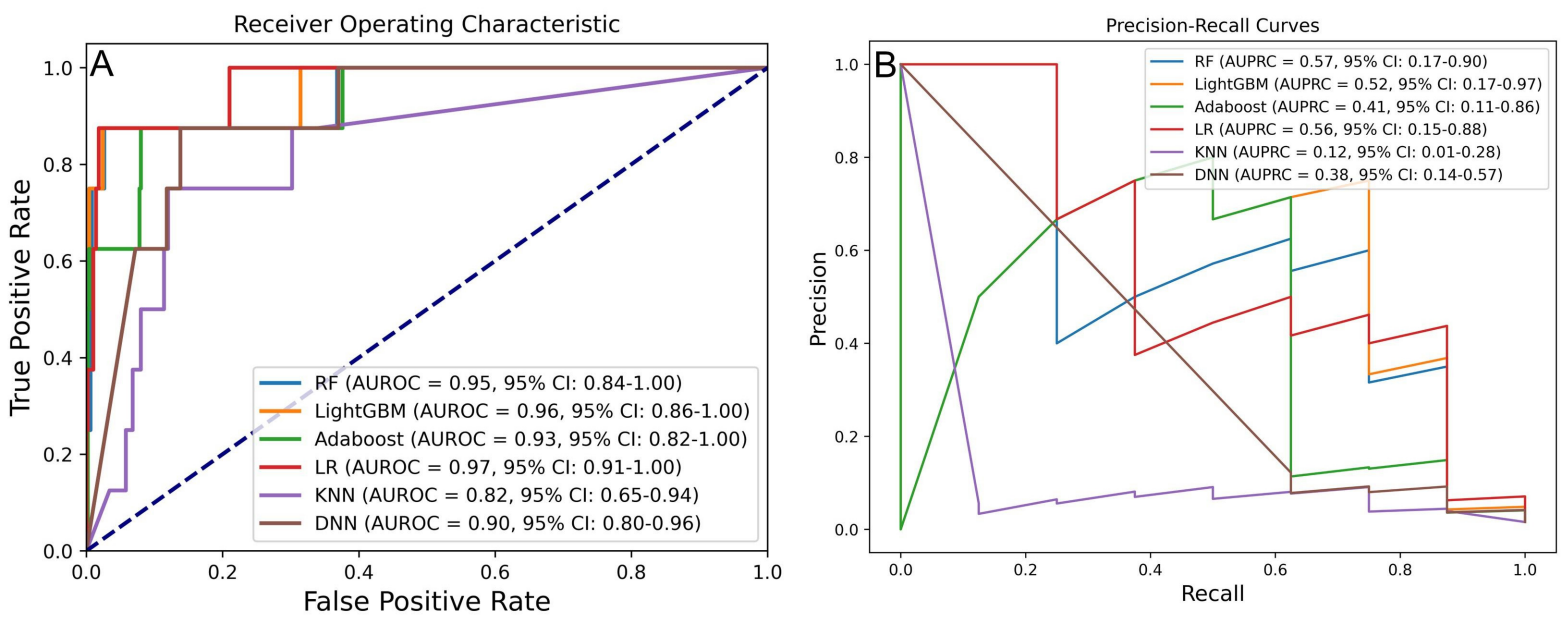
**Performance comparison of six ML models for predicting IHM in AMI post-PCI**. (A) AUROC and (B) AUPRC for models developed with different algorithms. RF, random forest; LightGBM, light gradient boosting machine; AdaBoost, adaptive boosting; LR, logistic regression; KNN, k-nearest neighbor; DNN, deep neural network.

The second phase aimed to identify the optimal ML algorithm using the previously determined best processing strategy. Six algorithms were evaluated, with the results of AUROC and AUPRC presented in Fig. 3A,B. Among these, RF, LightGBM, and LR demonstrated superior predictive performance. Specifically, RF achieved an AUROC of 0.95 (95% CI: 0.84–1.00) and an AUPRC of 0.57 (95% CI: 0.17–0.90); LightGBM reached an AUROC of 0.96 (95% CI: 0.86–1.00) and an AUPRC of 0.52 (95% CI: 0.17–0.97); LR recorded an AUROC of 0.97 (95% CI: 0.91–1.00) and an AUPRC of 0.56 (95% CI: 0.15–0.88).

DeLong and bootstrap tests were performed to compare AUROC and AUPRC differences across models (**Supplementary Fig. 2**). While the AUROC differences were not statistically significant (*p* > 0.05), RF and LightGBM significantly outperformed KNN in terms of AUPRC (*p* < 0.05).

Model evaluation using additional metrics—including accuracy, precision, sensitivity, specificity, and F1 score—further supported RF and LightGBM as the top-performing models. As shown in Table 3, RF achieved an AUROC of 0.948, an accuracy of 0.988, a precision of 0.625, a sensitivity of 0.625, a specificity of 0.994, and an F1 score of 0.625. LightGBM achieved an AUROC of 0.956, an accuracy of 0.992, a precision of 0.750, a sensitivity of 0.750, a specificity of 0.996, and an F1 score of 0.750.

**Table 3.** **Performance of six ML algorithms for predicting IHM in AMI patients post-PCI**.

Algorithms	Accuracy	Precision	Sensitivity	Specificity	F1-score	AUROC	AUPRC
RF	0.988	0.625	0.625	0.994	0.625	0.948	0.567
LightGBM	0.992	0.750	0.750	0.996	0.750	0.956	0.517
Adaboost	0.963	0.238	0.625	0.968	0.345	0.932	0.414
LR	0.571	0.035	1.000	0.564	0.068	0.967	0.564
KNN	0.825	0.065	0.750	0.826	0.119	0.821	0.119
DNN	0.933	0.139	0.625	0.938	0.227	0.916	0.376

### 3.3 Identification of the Final Model

**Fig. 4.**
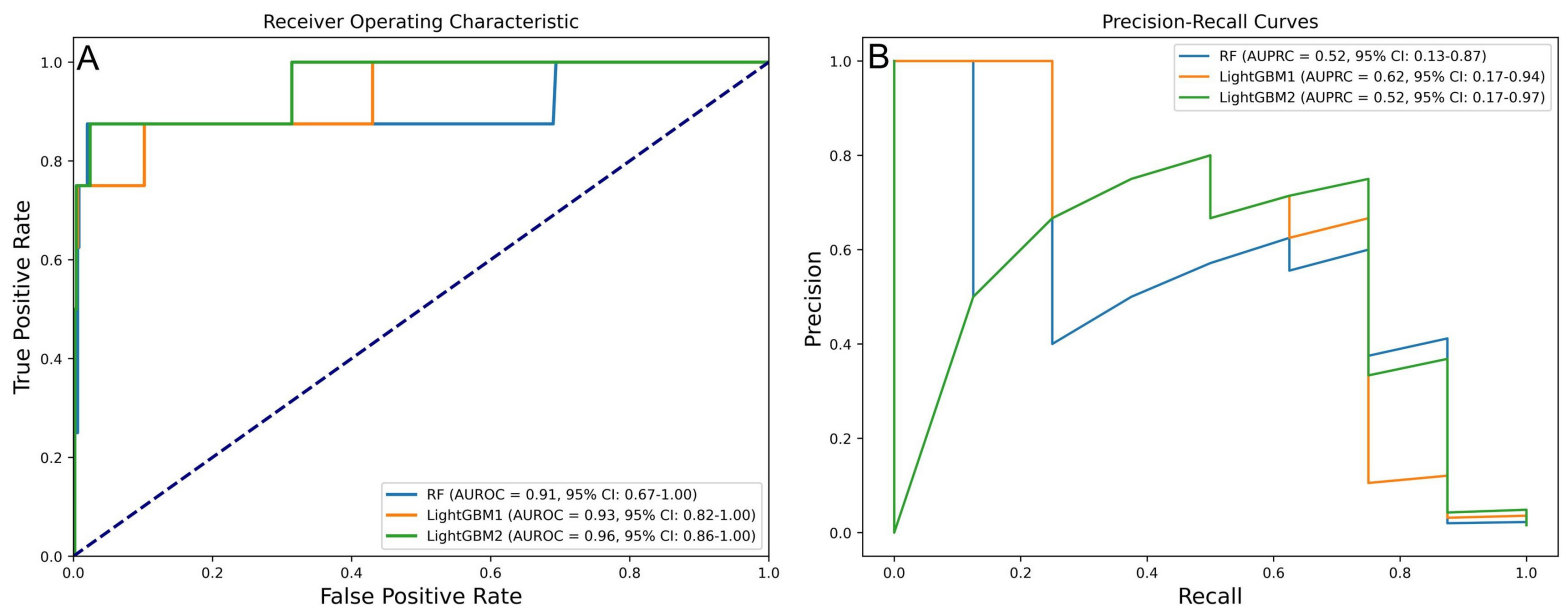
**Performance of three prospective models in predicting IHM post-PCI in AMI patients**. (A) AUROC values of prospective models. (B) AUPRC values of prospective models.

In the final model selection phase, the RF model, trained using SMOTE, Boruta, GSCV, and RF, was selected as the primary option. The LightGBM1 model, which utilized SMOTE, Boruta, GSCV, and LightGBM, was chosen as the secondary option, followed by the LightGBM2 model, which employed SMOTE, GSCV, and LightGBM. Key performance metrics—including accuracy, precision, sensitivity, specificity, F1 score, AUROC, and AUPRC—demonstrated significant improvements, as summarized in Tables 2,3. The AUROC and AUPRC analyses further underscored the strong performance of the LightGBM1 model (Fig. 4), which achieved an AUROC of 0.93 (95% CI: 0.82–1.00), an AUPRC of 0.62 (95% CI: 0.17–0.96), an accuracy of 0.988, a precision of 0.625, a sensitivity of 0.625, a specificity of 0.994, and an F1 score of 0.625.

**Fig. 5.**
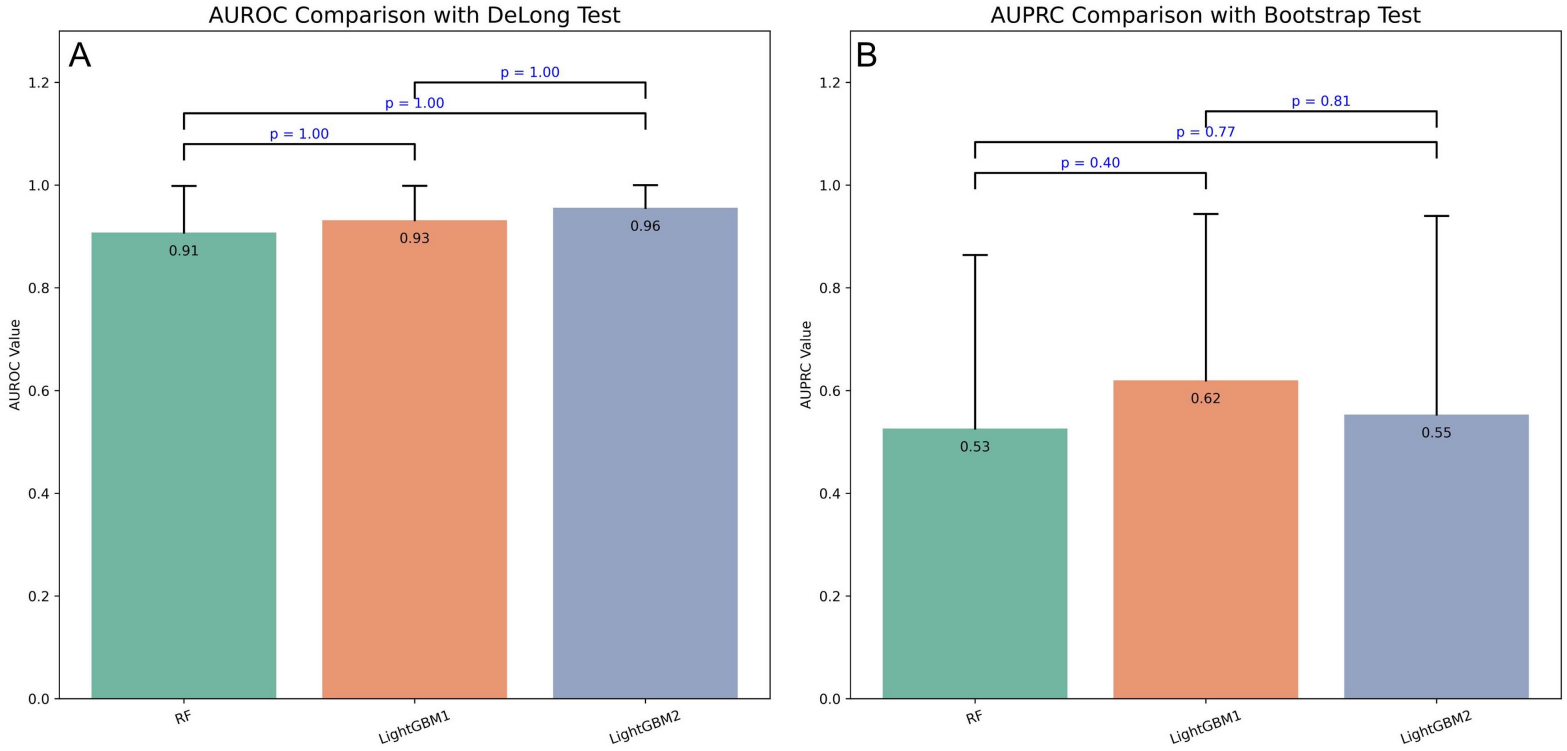
**Performance comparison of three prospective models using DeLong and bootstrap tests**. (A) DeLong test comparing AUROC values across three prospective models. (B) Bootstrap test comparing AUPRC values across three prospective models.

To compare model performance, DeLong and bootstrap tests were applied to the AUROC and AUPRC values (Fig. 5). Although LightGBM1 outperformed the other models on both metrics, the differences were not statistically significant (*p* > 0.05).

**Fig. 6.**
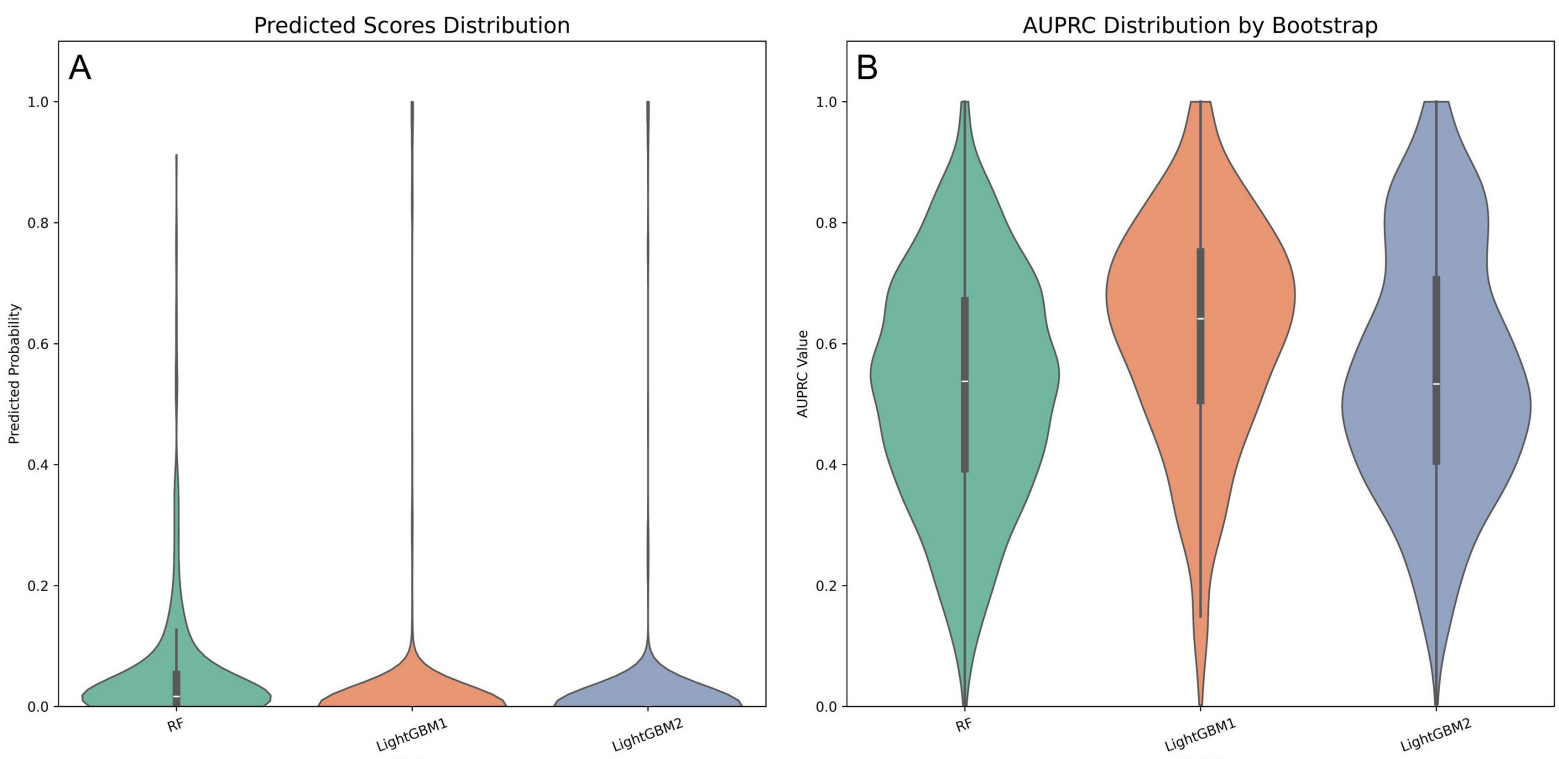
**Violin plots comparing performance metrics across three predictive models**. (A) AUROC distribution of three prospective models. (B) AUPRC distribution of three prospective models.

Violin plots were employed to visualize the probability density distributions of AUROC and AUPRC (Fig. 6). The LightGBM1 model exhibited a more concentrated AUROC distribution compared to LightGBM2 (Fig. 6A). Its AUPRC distribution was right-skewed, with a median around 0.7, indicating superior discriminative ability (Fig. 6B).

The results of the first phase demonstrated no statistically significant differences in model performance across the various processing methods. While the combination of SMOTE, Boruta, and GSCV effectively reduced feature dimensionality, it may have compromised predictive efficacy. Consequently, the final comparison focused on SMOTE combined with GSCV and SMOTE combined with Boruta and GSCV to identify the optimal processing approach. In the second phase, LightGBM exhibited a slight performance advantage over RF, although this difference was not statistically significant, which led to the inclusion of both algorithms in the final evaluation. In the final phase, based on comparisons of AUPRC and violin plots, the configuration of SMOTE, Boruta, GSCV, and LightGBM was identified as the optimal model configuration.

### 3.4 Model Development

**Fig. 7.**
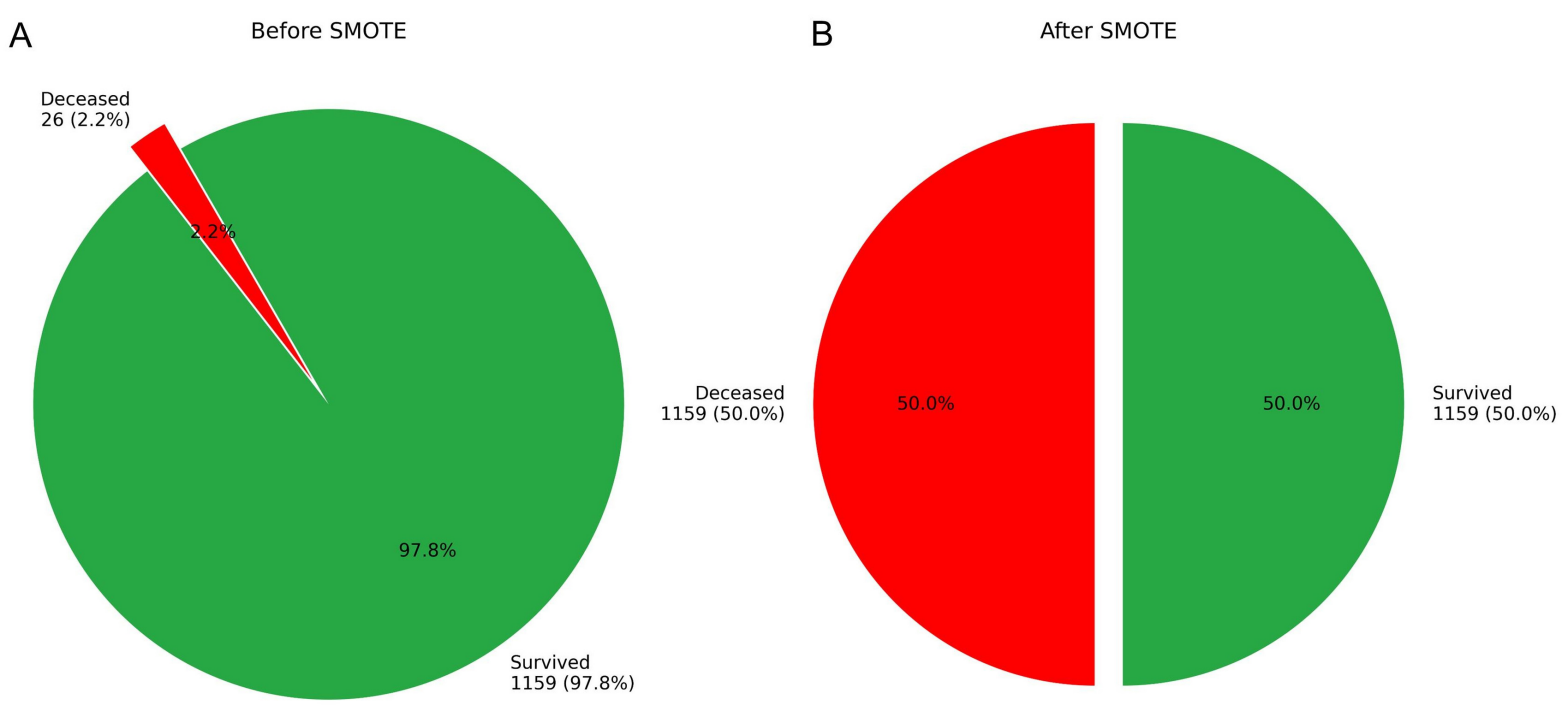
**Case distribution in the training cohort before and after SMOTE augmentation**. (A) Original distribution. (B) Post-SMOTE distribution.

The original dataset exhibited a significant class imbalance, which limited the effectiveness of various analytical algorithms. To facilitate robust model development, the class distribution was initially adjusted using the SMOTE, resulting in an increase in the number of deceased cases from 24 to 1159 (see Fig. 7).

To reduce model complexity and mitigate the risk of overfitting, feature selection was conducted utilizing the Boruta algorithm, which decreased the number of predictors from 39 to 32.

The optimized LightGBM model was configured with the following parameters: min_samples_split = 5 (the minimum number of samples required to split a node), n_estimators = 200 (the number of boosting iterations), and random_state = 3331 (to ensure reproducibility). These hyperparameters were selected through grid search to achieve a balance between model performance and computational efficiency.

### 3.5 Model Explanation

**Fig. 8.**
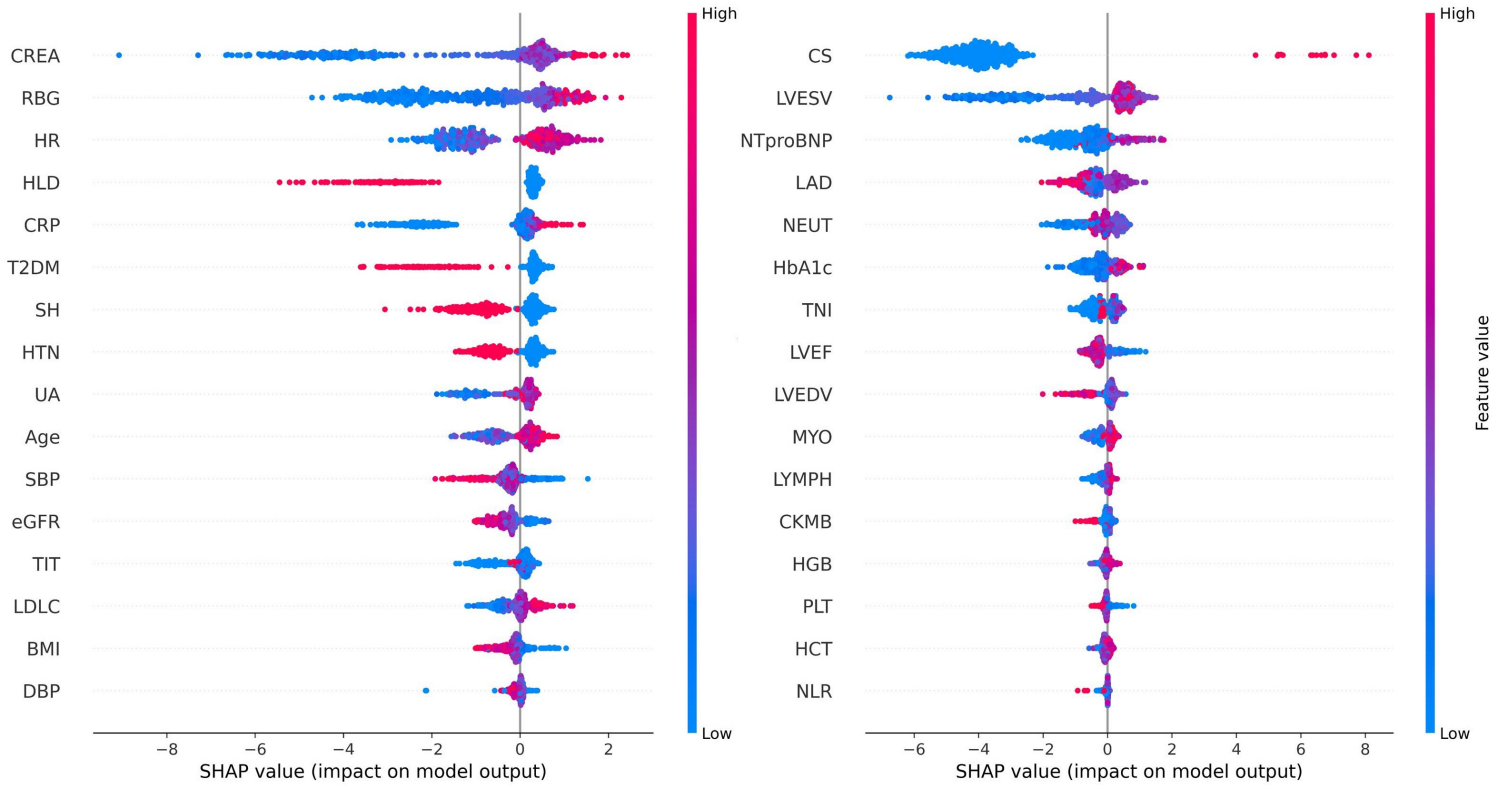
**SHAP summary plot for the LightGBM model predicting IHM in AMI patients post-PCI**. The plot shows each feature’s contribution to the model’s predictions.

To enhance clinical interpretability and address concerns regarding the explainability of ML models, SHAP was employed to elucidate the final model’s output by quantifying the contribution of each feature to individual predictions. SHAP summary plots illustrate feature importance in descending order based on mean SHAP values (Fig. 8). Each point represents an individual sample, with rows corresponding to specific features. The horizontal axis denotes SHAP values, while the color gradient (red indicating high values and blue indicating low values) reflects the original value of the feature.

**Fig. 9.**
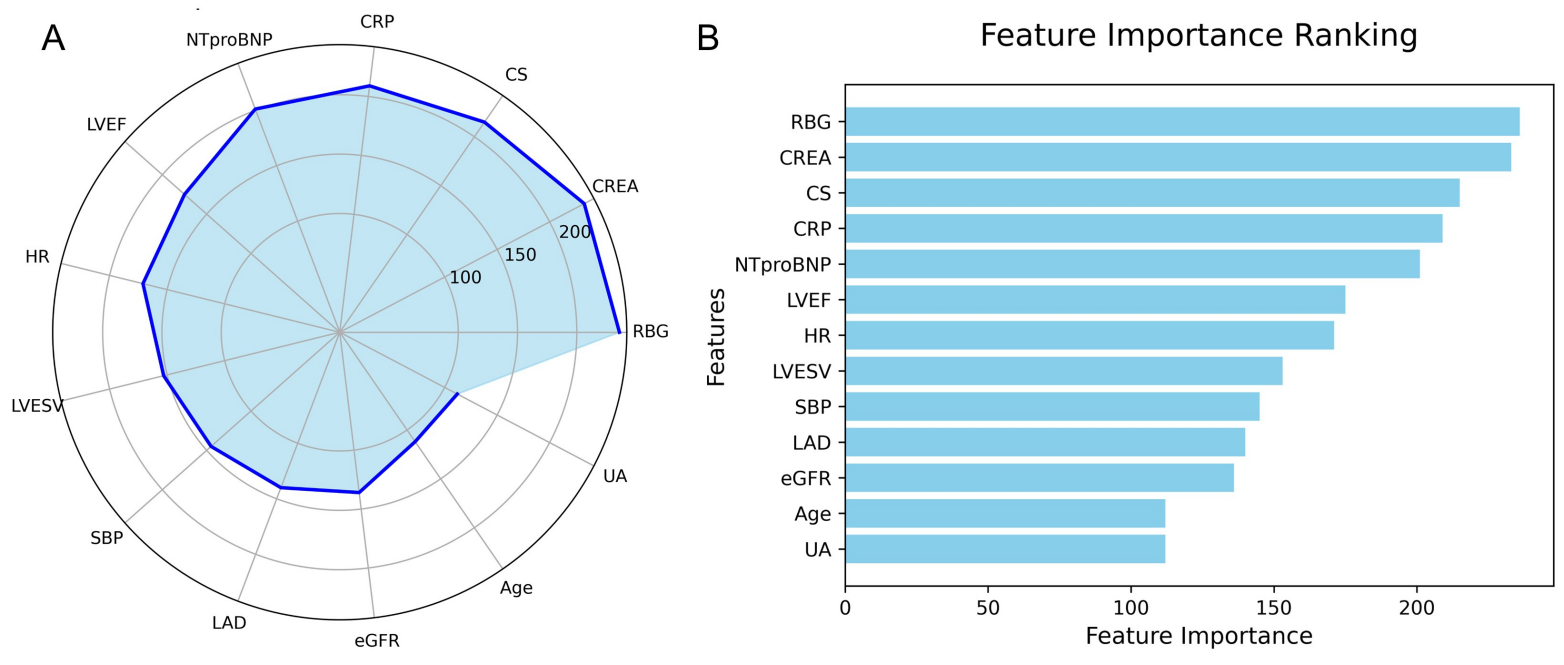
**Feature importance in the LightGBM model predicting IHM in AMI patients post-PCI**. (A) Radar plot of feature importance. (B) SHAP bar plot showing individual feature contributions.

The feature importance in the LightGBM model is visualized using a radar plot (Fig. 9A) and a SHAP summary bar plot (Fig. 9B). Both visualizations highlight the top 13 predictors of IHM, ranked by their relative contributions. RBG was identified as the most influential variable, followed by CREA and CS.

### 3.6 Clinical Utility and Web Application

**Fig. 10.**
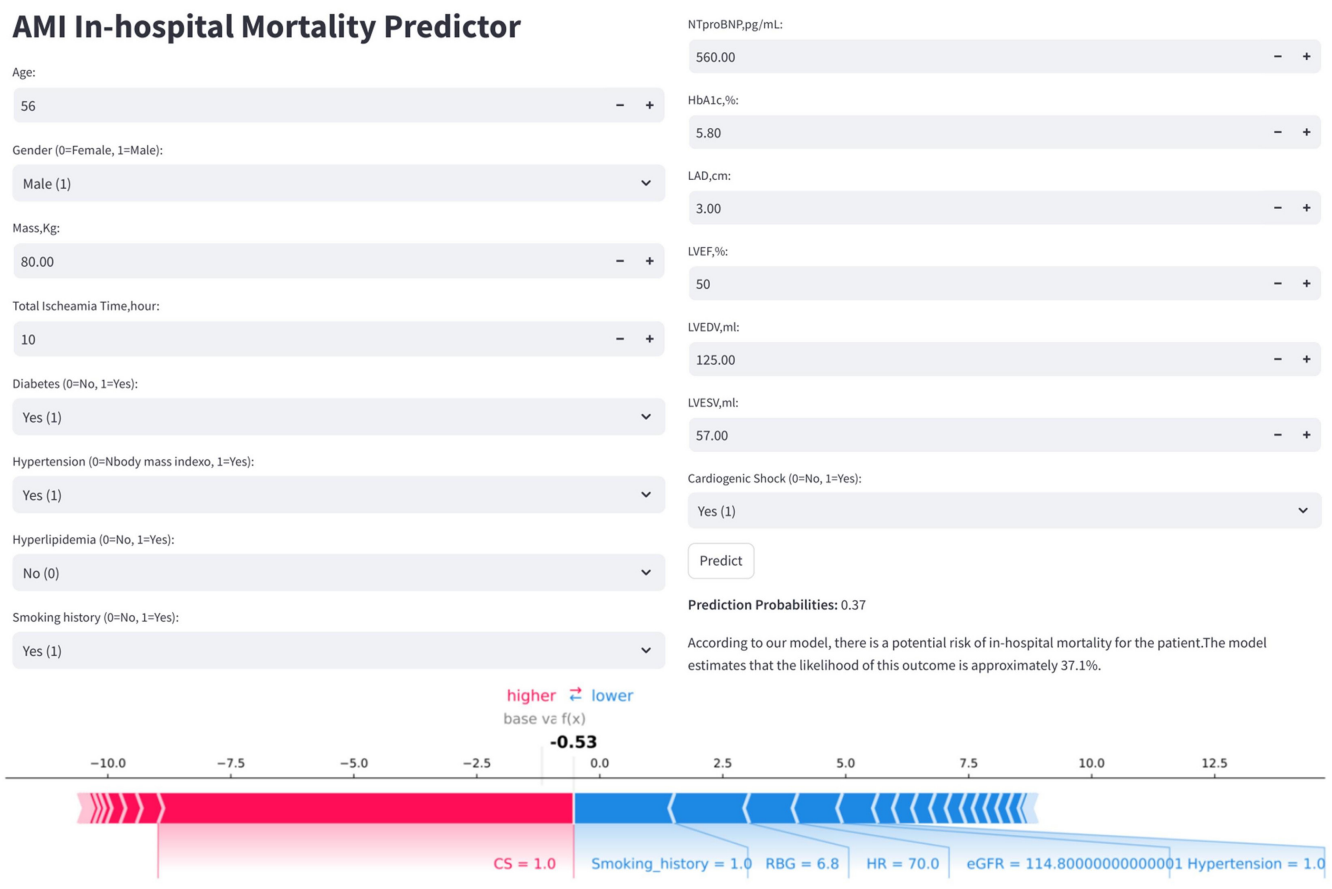
**Clinical application of the LightGBM model for IHM prediction in AMI patients post-PCI**. The application uses 32 input features to predict IHM probability (e.g., 37.1%). The accompanying SHAP force plot visualizes individual feature contributions: blue bars (right) push the prediction toward mortality, while red bars (left) favor survival.

To enhance the clinical applicability of the model, the final predictive model has been deployed as a web-based application (Fig. 10). Clinicians are able to input values for the 32 required features, and the application automatically estimates the individual risk of IHM for patients with AMI. Additionally, it generates a personalized SHAP force plot that visually highlights the factors influencing each prediction. In these plots, the blue features on the right indicate variables associated with improved survival, while the red features on the left represent factors that contribute to an increased risk of mortality.

## 4. Discussion

In our study, we developed a personalized IHM risk prediction model for patients with AMI following PCI, addressing the significant class imbalance in the dataset, which had an imbalance ratio of 49:1. To mitigate this imbalance, we employed the SMOTE, increasing the number of deceased cases from 24 to 1159. For feature selection, we utilized the Boruta algorithm, which identified 32 significant predictors from a total of 39 clinical indicators, thereby ensuring robust model performance and stability. Subsequently, we constructed and evaluated six ML algorithms utilizing the selected features. Among these algorithms, the ensemble methods exhibited superior predictive performance; for instance, LightGBM achieved an AUROC of 0.93 and an AUPRC of 0.62 on the validation set. To enhance interpretability, we applied the SHAP framework, which provided insights into variable importance and the underlying impact mechanisms of each predictor on the model’s output. Furthermore, we developed a web-based online calculator to facilitate the clinical adoption and dissemination of the model, enabling healthcare professionals to effectively assess IHM risk in AMI patients post-PCI.

Modeling data is the primary determinant of predictive model performance [22]. Our study underscores the necessity of data preprocessing when dealing with highly imbalanced datasets (imbalance ratio >5:1) [23]. Many predictive models prioritize classification accuracy without adequately addressing class imbalance, which results in a bias toward the majority class and poor detection of minority class outcomes [24, 25]. Khera *et al*. [26] conducted a comprehensive cohort study involving 755,402 patients hospitalized with AMI to evaluate the efficacy of ML models in predicting IHM. The overall IHM rate observed was 4.4%. Their findings indicated that ML models did not significantly enhance the discrimination of IHM compared to traditional methods, thereby limiting their clinical utility. Deng *et al*. [27] developed a ML model to predict no-reflow and IHM in STEMI patients undergoing primary PCI. Among the four algorithms tested, the RF model achieved the highest discrimination for IHM, with an AUROC of 0.9273, utilizing 37 predictors. The observed IHM rate was 5.5%. These studies involved datasets with significant class imbalance. While the models achieved high AUROC values, their low F1 scores indicate limited performance in identifying the minority class. This discrepancy suggests that the imbalance in the data may have constrained the algorithms’ effectiveness.

Feature selection is crucial in ML as modern datasets often contain an excessive number of predictors [28], many of which are irrelevant. Large feature sets can slow computations, waste resources, and diminish model accuracy. The minimal-optimal problem seeks to identify the smallest subset of features that maximizes classification performance, which has led to the development of numerous feature reduction algorithms [29, 30]. Emakhu *et al*. [31] analyzed a cohort of 31,228 patients, including 563 with ACS. Utilizing the Boruta feature selection method, they identified 11 significant risk factors. The resulting model demonstrated excellent predictive performance, achieving an AUROC of 93.3% and an F1-score of 86.3%. This study employed Boruta for feature selection, a robust, random forest-based method that compares original features with random ‘shadow features’ to identify key predictors, making it ideal for exploring biomarkers in complex datasets. We identified 32 significant predictors from the 39 features, with SHAP analysis highlighting 13 features that exhibited an importance value greater than 110, including RBG, CREA, CS, CRP, NTproBNP, LVEF, HR, LVESV, SBP, LAD, eGFR, age, and UA. RBG was strongly associated with poor in-hospital outcomes [32], while CS was closely linked to IHM [33]. Previous studies have also underscored the prognostic value of inflammatory markers such as WBC count and NEUTs in predicting IHM [34, 35, 36]. From a clinical perspective, while many of these predictors are associated with the prognosis of AMI, their importance can vary across different models. ML aims to identify the optimal combination of parameters and assign appropriate weightings to construct the most effective predictive model. If these predictors or cases do not adequately represent the overall population, the model may encounter challenges in achieving acceptance for broader application across diverse regions and populations.

We compared several representative ML algorithms to demonstrate their superiority over traditional statistical methods [37, 38]. These algorithms included RF, LightGBM, AdaBoost, LR, KNN, and DNN. LR served as the classical statistical benchmark, while DNN was selected to represent deep learning. Although KNN is among the simplest ML algorithms, it struggles with computational efficiency, particularly in high-dimensional datasets. In contrast, DNNs excel at identifying complex patterns through deep layers of abstraction, making them well-suited for tasks such as image recognition and natural language processing. However, DNNs often present the ‘black-box’ problem, which makes ensemble methods like RF and LightGBM more interpretable and thus popular choices in medical ML.

AUROC is a widely used metric for evaluating binary classifiers, as it measures a model’s ability to discriminate between positive and negative cases across all thresholds. While both AUROC and AUPRC assess the separation of risk scores, AUPRC places greater emphasis on positive cases, whereas AUROC treats all cases equally. Given that AUROC can be misleading in imbalanced datasets, this study prioritized AUPRC for more reliable model selection. Although many studies have developed similar IHM models using only a limited number of features, these models typically exhibit lower class imbalance ratios than ours and prioritize AUROC over AUPRC. These factors may account for the relative complexity of our model. In the future, we plan to leverage web-crawling technology to integrate our web application into hospital information systems (HIS) to facilitate easier clinical implementation.

Most conventional AMI prediction models are designed for population-based risk stratification, categorizing patients into broad risk groups (low, medium, high). Consequently, clinical guidelines are often population-based and not directly applicable to individual cases. In contrast, ML models enable patient-specific predictions [39], facilitating personalized treatment plans and marking a shift from population-based to individualized care. This personalized approach can improve clinician decision-making efficiency and enhance patient-provider interactions.

## 5. Limitations

Our study has several limitations. First, it is a single-center, retrospective analysis with a relatively small sample size, underscoring the need for larger, multicenter prospective studies. Second, the absence of external validation limits the assessment of the model’s generalizability. Third, we evaluated only six ML models, which may constrain the comprehensiveness of our findings. Finally, while Boruta was utilized for feature selection, the resulting model remains complex, and no direct comparisons were conducted with other feature selection methods.

## 6. Conclusion

In conclusion, we employed SMOTE for class balancing, Boruta for feature selection, GSCV for optimal hyperparameter tuning, and LightGBM for model development. This approach resulted in the creation of a ML tool designed to predict IHM in AMI patients following PCI within a clinical database, while addressing issues of dimensionality reduction and class imbalance. These findings underscore the importance of robust data processing and careful algorithm selection.

## Availability of Data and Materials

The datasets used and/or analyzed during the current study are available from the corresponding author on reasonable request. The source code for the predictive model used in this study is publicly available at the following GitHub repository: https://github.com/gralearn/IHM_Predictor.git.

## Supplementary Material

Supplementary material associated with this article can be found, in the online version, at https://doi.org/10.31083/RCM39271.
