# Supplementary material for: Machine Learning Approach on Predictive Model Establishment for In-Hospital Mortality in Acute Myocardial Infarction Patients Post-Percutaneous Coronary Intervention: Solutions for Databases With Dimensionality Reduction and Class Imbalance
Source: Rev Cardiovasc Med. 2025 Sep 25;26(9):39271. doi: 10.31083/RCM39271 (PMC12516744; doi:10.31083/RCM39271)
Supplement: Supplementary file 1 [file 2153-8174-26-9-39271-s1.docx]

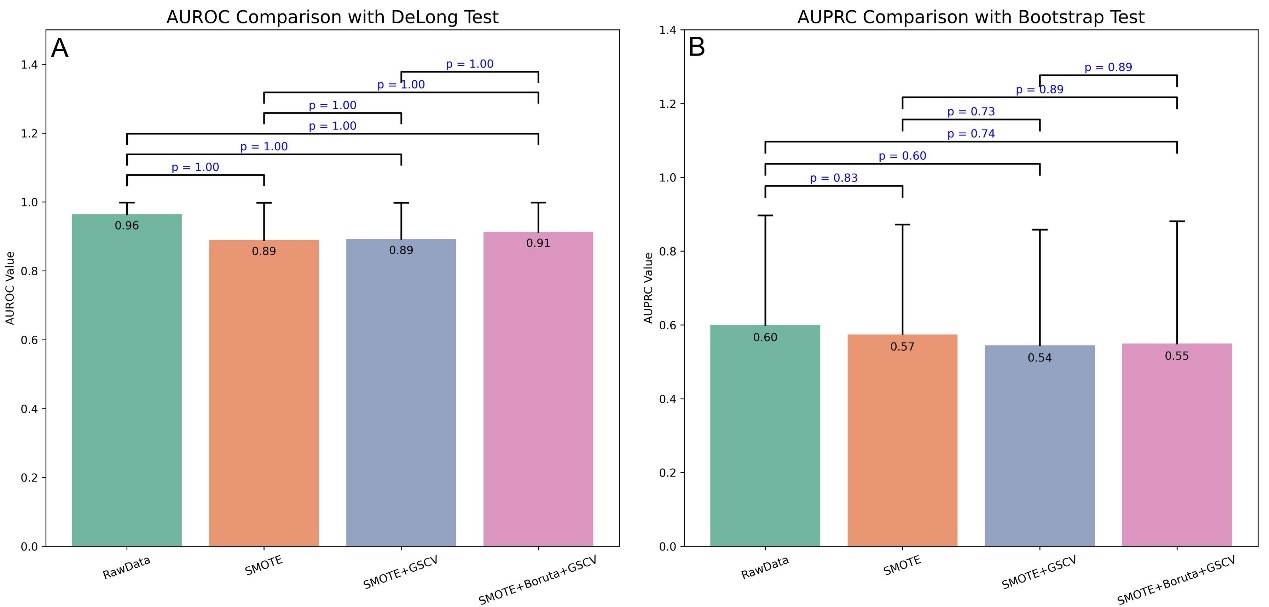


**Supplementary Fig. 1. Statistical comparison of model performance under different data processing strategies using DeLong and bootstrap tests. (A) DeLong test comparing AUROC values across models. (B) Bootstrap test comparing AUPRC values across models.** SMOTE, synthetic minority over-sampling technique; GSCV, grid search with cross-validation; AUROC, area under the receiver operating characteristic curve; AUPRC, area under the precision-recall curve.


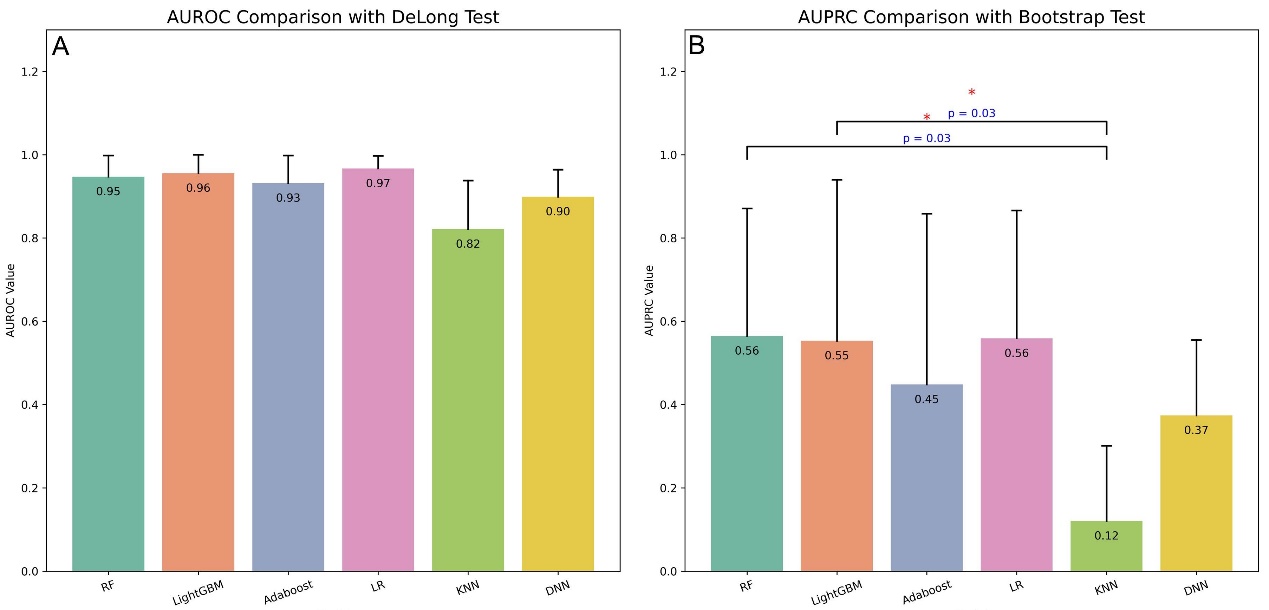


**Supplementary Fig. 2. Impact of ML algorithms on model performance, evaluated using DeLong and bootstrap tests. (A) DeLong test comparing AUROC values across different ML models. (B) Bootstrap test comparing AUPRC values across different ML models.** ML, machine learning; RF, random forest; LightGBM, light gradient boosting machine; AdaBoost, adaptive boosting; LR, logistic regression; KNN, k-nearest neighbor; DNN, deep neural network; AUROC, area under the receiver operating characteristic curve; AUPRC, area under the precision-recall curve.
